# Regional Initiatives in the Western Hemisphere as a Contribution to the Safe Biotechnology Development

**DOI:** 10.3389/fbioe.2022.837635

**Published:** 2022-02-04

**Authors:** Pedro J. Rocha-Salavarrieta

**Affiliations:** Inter-American Institute for Cooperation on Agriculture (IICA), San José, Costa Rica

**Keywords:** biosafety, biotechnology regulation, institutionalism, GMOs, gene editing, regulatory cooperation

## Introduction

Under the spirit of collaboration and coordination, countries have created several instruments to address biotechnology and biosafety (B&B) issues. For instance, the CBD (United Nations, 1992), the CPB (Secretariat of the Convention on Biological Diversity, 2000), and the Codex Alimentarius guidelines on risk assessment (FAO, 2021). In addition, neighboring countries have reached some agreements to consider B&B issues from a regional perspective.

Whether global or regional, such instruments establish general guidelines that seek similarity in the treatment of certain issues or the application of specific requirements. For example, not to affect transboundary movement or trade, taking advantage of technological developments, assessing risks in an objective manner, promoting food safety and quality, and achieving global environmental sustainability, thus favoring comprehensive and safe development.

## Group 5 of the Agricultural Council of the South (G5-CAS)

The Agricultural Council of the South (CAS, for its name in Spanish), created in April 2003, is integrated by the ministers of agriculture of Argentina, Bolivia, Brazil, Chile, Paraguay, and Uruguay. It is a forum for consultation and coordination of regional actions, whose purpose is to define the priorities of the agricultural agenda and take positions on issues of regional interest in order to coordinate specific actions (CAS, 2021). The CAS hold regular meetings and presents very concise and pragmatic “Ministerial Declarations”.

To identify short- and medium-term joint actions for regional cooperation, the CAS has the Agricultural Policy Coordination Network (REDPA) that embraces the Directors of Agricultural Policies and its various Technical Groups, including Technical Group 5 (G5-CAS) on Public Policies on Biotechnology. The G5-CAS includes national experts from five of the six CAS countries (except Bolivia) who analyze different topics, then generate regional position proposals on strategic B&B issues, according to the needs of the region, for Council’s ministers debate and approval.

G5-CAS recognized the importance of genome editing (GnEd) for agriculture development, the need for having science-based decisions to promote research and development, and to avoid non-justified barriers to international trade. Based on that, the ministers agreed on fostering the technology; calling in different international fora for the application of transparent science-based regulatory frameworks; to promote capacity building activities; and to encourage the collaborative work for exchanging information about products development and regulatory advances (Table 1). This clear institutional support to the technology explains, in part, the technical and regulatory advances on GnEd in the Southern Cone and its encouragement to other countries and regions.

**TABLE 1:** Some statements issued by G5-CAS.

Ministerial declaration (date)	Statement
XXXVII-2019 (28–29/05/2019)	Statement III. Low level presence of genetically modified organisms not authorized by the importing country. (LLP)
	http://consejocas.org/wp-content/uploads/2019/05/XXXVII-RO-CAS-Declaraci%C3%B3n-III.-Low-Level-Presence.pdf
XXXVI-2018 (20–21/09/2018)	Statement I. Access to third markets for GMO products and their derivatives
	http://consejocas.org/wp-content/uploads/2018/09/XXXVI-RO-CAS-Declaraci%C3%B3n-I.-Acceso-a-terceros-mercados-de-productos-OGM-y-sus-derivados.pdf
	Statement II. Genome Editing Techniques
	http://consejocas.org/wp-content/uploads/2018/09/XXXVI-RO-CAS-Declaraci%C3%B3n-II.-T%C3%A9cnicas-de-Edici%C3%B3n-G%C3%A9nica.pdf
XXXV-2018 (3–4/05/2018)	Statement I. Priorities of the Agricultural Council of the South
	Opening to third markets of biotechnology events in the region, such as GMOs and NTBs
	http://consejocas.org/wp-content/uploads/2018/05/Declaraci%C3%B3n-I.pdf
XXXIV-2017 (27/08/2017)	Statement III. New breeding techniques and access of GM products to third markets
	http://consejocas.org/wp-content/uploads/2017/08/Declaracio%CC%81n-III-Nuevas-tecnologi%CC%81as-de-mejoramiento-y-acceso-de-productos-GMs-a-terceros-mercados-1.pdf
XXXII-2016 (3–4/11/2016)	Statement III. Negotiation of the Cartagena Protocol (COP-MOP8)
	http://consejocas.org/wp-content/uploads/2016/11/Declaracio%CC%81n-III-Negociacio%CC%81n-del-Protocolo-de-Cartagena.pdf
	Statement IV. Development of new breeding technologies
	http://consejocas.org/wp-content/uploads/2016/11/Declaracio%CC%81n-IV-Desarrollo-de-Nuevas-Tecnologi%CC%81as-de-Mejoramiento.pdf

## North American Biotechnology Initiative (NABI)

The NABI, signed in October 2003, was a high-level policy dialogue on topics related to agricultural biotechnology for regulators from Canada, Mexico, and United States. Its objectives were to exchange information among members, discuss common interest topics, and promote the development of innovative and cooperative approaches in order to regulate products of agricultural biotechnology as well as identify areas for further cooperation ranging from scientific research, collaborations, market access, and regulatory regimes.

Remarkably, NABI reached the trilateral arrangement on the “Documentation Requirements for Living Modified Organisms for Food or Feed, or for Processing (LMO/FFP’s)”, an important mechanism that allowed the implementation of Article 18.2 (a) of the CPB. Apart from facilitating Mexico to accomplish its obligations to the CPB without disrupting intra-regional trade, this arrangement ensured certainty in the trading environment between parties and non-parties of CPB (Winkles, 2004), which has been demonstrated, as global trade of LMOs continues today based on this arrangement.

## Initiative for Central America in Biotechnology and Biosafety

The ICABB, created in March 2013, is a platform for dialogue and technical exchange on issues of interest in agricultural B&B (IICA, 2013). It comprises the coordinators of the National Technical Commissions of Biosafety of Belize, Costa Rica, El Salvador, Guatemala, Honduras, Nicaragua, Panama, and the Dominican Republic.

Operationally, ICABB organizes meetings to present regulatory advances, propose training, establish communication activities, and analyze documents in order to fulfill its consultative function on biosafety issues for the countries of the region. Some ICABB achievements include a workshop on risk assessment (IICA/UNEP-GEF, 2013) and the review of a technical document -proposed by one of its members-that indirectly contributed to both the generation of a national biosafety regulation and the support to the customs union agreement explained further down.

## Customs Union Agreement El Salvador-Guatemala-Honduras

The customs union agreement between Guatemala and Honduras is a form of trade integration, operating since June 2017 (SIECA/CEIE, 2018), and expanded with El Salvador in August 2018 (SICA, 2021). This instrument is a deep integration process, led by a ministerial committee of the three member States that promotes free transit of goods and services. Among many other issues, the ministerial committee has discussed and taken decisions on the use of B&B for the agricultural sector.

The tri-national group proposed the “Technical Rule on Biosafety of Living Modified Organisms for Agricultural Use, RT65.06.01:18″ through a strict process of technical discussions and formal protocols (for regular meetings; participation of different agencies-agriculture, environment, and economics-; and public consultations, both national and international).

Interestingly, although this rule is a multinational instrument, ratified and implemented by each country, it has not displaced national legislations, but on the contrary, it complemented them by providing technical and legal support for the revision (Honduras) and generation (Guatemala) of their biosafety regulatory frameworks, offering greater technical, operational, and administrative clarity (SIECA, 2019).

Consequently, Honduras generated and approved the authorization procedure for applications related to the use of precision biotechnology (The Gazette Official Journal of Honduras, 2019). Guatemala created its Technical Committee of Agricultural Biotechnology (named CTBAG; Central American Journal of Guatemala, 2019), and established its biosafety legal framework of LMOs that includes specific provisions addressing the regulatory status of GnEd products (Central American Journal of Guatemala, 2019a). Due to its subsequent integration, El Salvador advances in the discussion and the eventual issuance of a biotech regulation for agriculture.

Therefore, through transparent, predictable, and rigorous regulatory B&B national systems, the customs union agreement strengths the agricultural sector, reinforces the national institutionality, provides new opportunities for developers, and offers farmers access to biotechnological alternatives.

## Discussion

In a global scenario characterized by complex commercial, social, political, legal, technological, productive, and environmental dynamics, the relationship and negotiation between countries are imperative, and lead to the promotion of multilateral cooperation. Due to heterogeneity among countries, complete harmonization of laws or standards related to LMOs will probably not be possible, but regulatory cooperation is an effective alternative in that direction.

Regulatory cooperation through regional initiatives/platforms in B&B helps to optimize the technical resources available in the countries and regions, allowing to identify potential conflicts and, more importantly, to determine possible ways to resolve them [e.g. NABI and Art.18.22 (a) of CPB]. In addition, their agile and informal governmental schemes (characterized by administrative flexibility and technical rigor) contribute to the optimization of decision-making. Regional initiatives provide more clarity, transparency, and confidence in the assessment systems and institutions, opening the door to the use of common (and simplified) procedures based on the recognition of third countries assessments when justified (e.g. Paraguay) as well as considering the evaluations and regulatory decisions of peers in other countries. With that, work duplications avoid, resources optimize, and response times accelerate, which is relevant for international trade.

The nonbinding decisions taken in regional initiatives offers important technical orientations and policy references for national regulations, which have caused positive impact on biotechnology access, technology transfer, and product commercialization. In addition, such platforms encourage and guide other countries and regions. For instance, CAS and NABI stimulated ICABB creation, and the later contributed some elements for the technical rule of the Customs Union Agreement. In this manner, regional initiatives show both possible options and practical pathways for addressing current and future biosafety issues in a very articulate way.
